# Injuries to the Spine

**Published:** 1894-02

**Authors:** J. Y. H. Smith

**Affiliations:** Rochelle, Ga.


### INJURIES TO THE SPINE.

BY J. Y. H. SMITH, M. D.,
ROCHELLE, GA.

### REPORT OF TWO CASES.

On October 5th I was called to see J. G., mill hand, colored.

*Diagnosis:* Injuries to spinal cord, with hemorrhage in upper lumbar region.

*History:* During a dispute the patient was stabbed in the back with small blade of a knife. He fell to the ground, and on attempting to rise found he could not use one leg.

On examination I found complete motor paralysis, but no paralysis of sensation. Under palliative treatment patient began to improve in a few days, and in about six weeks regained complete use of limb.

SECOND CASE: W. J. L., age thirty-five years. Printer by trade. I was called to see him on December 25th, 1893. Diagnosed pressure on the spine. Most probably caused by hemorrhage.

*History:* After retiring at a late hour on Saturday night, 23d, in an intoxicated condition, patient got up and went to printing office, and said he fell from top of stairway to the ground, a distance of fifteen feet. He was found early next morning unable to rise, and had to be carried to his room. I was called a few hours later.

On examination, found complete paralysis from upper dorsal region down, both motor and sensation; had very little use of arms and no use of fingers. Said they felt like they were asleep. Could not feel the prick of a pin in his feet. Had not passed his water since the fall. Bowels had not moved. He suffered no pain except on being moved, and then at the lower cervical and upper dorsal region. Pulse 84; respiration 15; temperature normal. Patient had been stimulated with whisky since fall and was not free from influence of it when I reached him. I relieved his bladder, and gave thirty drops fluid extract of ergot every three hours, and four grains phenacetine every four hours. During the day pulse went to 90; respiration 18.

On 26th patient was about free from stimulants. Pulse 90; temperature 99½°. In the evening of same day, pulse 100; temperature 104¾° F. After drawing his water gave him several enemas, until bowels acted well, and through the night gave twenty grains antifebrin.

On morning of 30th his symptoms had apparently improved. Pulse 95; temperature 100°. Said he had slept well during the night, and had taken nourishment. Patient had lain on back since the fall, and when placed in any other position experienced such pains as to render it impossible for him to remain in it. Had him turned on side and spine rubbed several times with liniment. In the evening temperature was 103° axilla, pulse 70. Showed signs of pulmonary œdema and died early in the evening.

Sixteen hours later Dr. G. H. Macon made an autopsy and found a dislocation between third and fourth dorsal vertabræ with hemorrhage into the spine.

---

### SYPHILIS AND PREGNANCY.

Fournier (*Gazette des Hopitaux*) believes that two of the most important factors in the diagnosis of hereditary syphilis in a family are great frequency of abortion and high infantile mortality. Abortion is least frequent when the father alone is syphilitic, more frequent when the mother alone is syphilitic, and most constant when both parents are infected. In the latter cases as many as nineteen abortions have been known to occur. Fournier attended a family in which the first three children were all born at term and all robust. Then the father contracted syphilis, and his wife became infected; she aborted three times in succession. Fournier found that at the Lourcine Hospital one hundred and forty-five out of one hundred and sixty-seven of the children born of syphilitic mothers died in the institution. Collecting trustworthy statistics of four hundred and forty-one cases reported elsewhere, one hundred children whose mothers were syphilitic survived infancy, while three hundred and forty-one died. It is noteworthy that out of the three hundred and forty-one that died, three hundred and thirty-five perished within their first year; only six died later. Out of nine children in a syphilitic family, only two are likely to survive their first year.—*Canada Medical Record.*

